# Field-Programmable Gate Array-Based Chaos Oscillator Implementation for Analog–Discrete and Discrete–Analog Chaotic Synchronization Applications

**DOI:** 10.3390/e27040334

**Published:** 2025-03-23

**Authors:** Ruslans Babajans, Darja Cirjulina, Deniss Kolosovs

**Affiliations:** Institute of Photonics, Electronics and Telecommunications, Riga Technical University, 6A Kipsalas Street, LV-1048 Riga, Latvia; darja.cirjulina@rtu.lv (D.C.); deniss.kolosovs@rtu.lv (D.K.)

**Keywords:** chaos, chaotic modeling, chaos oscillator, chaotic synchronization, field-programmable gate arrays

## Abstract

This work focuses on evaluating the behavior of analog chaos oscillators in field-programmable gate arrays (FPGAs). This work is motivated by a new approach to designing chaos-based communication systems using chaos oscillator circuits implemented in hardware in the transmitter and the mathematical models of the oscillator implemented on an FPGA in the receiver. Such a hybrid approach opens new possibilities for chaos-based modulation schemes for wireless sensor network (WSN) applications. This work brings a hybrid chaos-based communication system closer to realization by implementing the chaos oscillators on an FPGA and achieving analog–discrete and discrete–analog chaotic synchronization. First, this paper derives a model that simulates the dynamics of Vilnius and RC chaos oscillators using Euler–Cromer numerical integration in fixed-point arithmetic. The derived MATLAB model precisely describes the digital design and is thus directly transferred to VHDL. The synthesized digital design is compiled onto an FPGA chip and is then used to achieve analog–discrete and discrete–analog Pecora–Carroll chaotic synchronization.

## 1. Introduction

The increasing application of smart interconnected devices in the Internet of Things (IoT) has led to the transformation of various industry sectors. While the primary motivation for this development is to facilitate control, data acquisition, and processing for industrial, medical, agricultural, and many other applications, it is also driven by advances in cellular network technology like 5G. According to the Ericsson mobility report [1], 5G is set to increase the range of IoT use cases, and the number of IoT connections is forecast to surpass 7 billion by 2030.

The increase in data transmission and reliance on interconnected devices poses security challenges. In [2,3], the authors described the taxonomy of security attacks on the IoT, breaking them down into physical, network, and application layers. Over the years, the security question has been addressed by introducing techniques such as encryption, blockchain, and cloud computing [4]. However, these techniques mainly covered the network and application layers, and data transfer security on the physical layer has received considerably less attention. One possible solution is the use of chaos phenomena to enhance communication security.

Chaotic systems are characterized by their sensitivity to initial conditions, wide spectrum, and noise-like waveforms. However, chaotic systems are deterministic, meaning that the system’s dynamics are not random like noise and thus can be exploited controllably. Chaotic systems are based on nonlinearity and can have different physical implementations. In the literature, chaos oscillators are low-power analog circuits that exhibit chaotic behavior and are deliberately designed to act as a source of chaotic signals. The first widely studied chaos oscillators were the Chua chaos oscillator [5], the Colpitts chaos oscillator [6], the Vilnius chaos oscillator [7], the RC chaos oscillator [8], and the Memristor chaos oscillator [9]. Over the years, some authors have introduced modifications to the original circuits [10,11,12], and other circuits have been proposed [13,14,15,16]. Chaos oscillators consist of inductors, capacitors, resistors, diodes, transistors, and operational amplifiers. Chaotic behavior can also be seen in other circuits under specific conditions, such as DC-DC converters, as demonstrated in [17,18].

Regarding discrete chaotic sources, the most common approach is chaotic maps—discrete nonlinear functions. Chaotic maps are commonly used for encryption due to their straightforward, discrete nature [19,20,21,22]. Another way of implementing discrete chaos is by acquiring numerical solutions to nonlinear circuit equations. Although this is a novel approach, it has gained popularity in recent years [23,24] due to better control over initial conditions and mitigation of imprecision of analog components. Discrete chaotic sources have also gained popularity due to the possibility of implementing them in field-programmable gate arrays (FPGAs), as demonstrated in [25,26,27,28,29,30,31,32].

Data transmission security on the physical layer can be addressed by using chaotic signals as carriers. Many chaos-based modulation schemes have been introduced, which can be divided into coherent and non-coherent schemes [33]. Coherent schemes require chaotic synchronization [34,35]; the two chaotic sources are asynchronous due to sensitivity to the initial conditions. Non-coherent schemes do not require chaotic synchronization [36,37]. The key feature of coherent schemes is that data recovery in the receiver is possible only using synchronized sources.

Previously, modulation schemes were designed to use either analog hardware circuits or chaotic maps. Our previous work [38] suggested a hybrid communication system that utilizes low-power analog chaos oscillators at sensor nodes and discrete chaos oscillators in the gateway. The core of such a system is the possibility of analog–discrete and discrete–analog chaotic synchronization. In [38], we demonstrated the possibility of this synchronization in the case of Vilnius and RC chaos oscillators. However, the discrete chaotic oscillator was modeled and implemented only in MATLAB. The current work further develops this system by implementing discrete chaos oscillators on an FPGA and exploring analog–discrete and discrete–analog chaotic synchronization. To summarize, the key contributions of this work are as follows:

- We implement discrete chaos oscillator models in fixed-point arithmetic for FPGA applications;
- We experimentally demonstrate the possibility of chaotic synchronization of the chaos oscillator circuit and discrete model implemented on an FPGA.

Studies centered on chaotic synchronization mainly focus on synchronization techniques for analog chaos oscillators or discrete chaotic maps [39,40,41,42]. In [43,44,45,46,47], the authors focused on the application of chaotic synchronization, while the authors of [25,26,27,28,29,30,31,32] presented the implementation of chaos on FPGAs. To the best of our knowledge, the only notable works that have explored the possibility of synchronizing the analog chaos oscillator and the discrete model of the oscillator are [23,48]. However, the authors only explored the analog–discrete synchronization case. The novelty of this work lies in the implementation and verification of FPGA-based analog–discrete and discrete–analog chaotic synchronization.

This paper is structured as follows. Section 2 details the steps of implementing Vilnius and RC chaos oscillators on an FPGA. It starts with the chaos oscillators and their state-variable differential equations, followed by the discrete implementation of these equations in fixed-point arithmetic, which leads to hardware implementation and the derivation of an accurate model. Section 3 verifies the possibility of chaotic synchronization between chaos oscillators implemented in circuit form and their FPGA-based counterparts. Section 4 studies the noise immunity of chaotic synchronization by comparing analog–discrete and discrete–analog synchronization methods. Section 4 concludes this work.

## 2. Chaos Oscillator FPGA Implementation

This section describes the FPGA implementation of chaos oscillators. It also describes the chaos oscillators, including their theory of operation through state-variable differential equations, followed by crucial details and design solutions for their digital implementation. In addition, it provides the implementation details for the Vilnius [7] and RC [8] chaos oscillators; however, the approach can also be applied to other chaos systems.

### 2.1. Vilnius Chaos Oscillator

**Figure 1:**
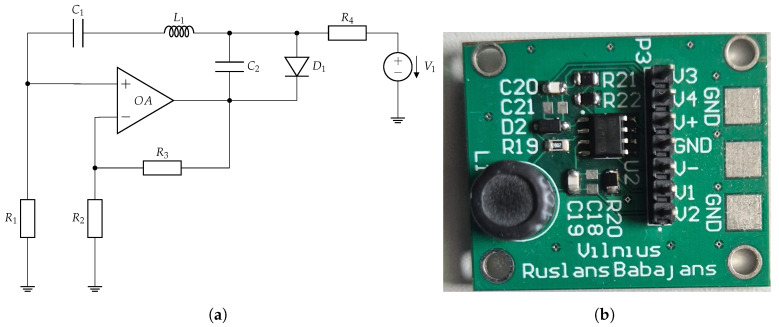
Vilnius chaos oscillator [7] (**a**) and PCB (**b**), with the following parameters: $C_{1}=1$ nF, $C_{2}=150$ pF, $L_{1}=1$ mH, $R_{1}=1$ kΩ, $R_{2}=10$ kΩ, $R_{3}=6$ kΩ, $R_{4}=20$ kΩ, and $V_{1}=5$ V. On the PCB, OA is an LT082 operational amplifier, and the diode is a 1N4148.

The first oscillator used in this work is the Vilnius chaos oscillator [7]. The circuit of the oscillator is shown in Figure 1a, while the hardware implementation of the circuit, using discrete components on an FR4 PCB, is shown in Figure 1b.

The dynamics of this oscillator are described using differential equations in (1). The state variables are the voltages across $C_{1}$ and $C_{2}$ and the current through $L_{1}$.

(1) $$\left\{\begin{array}{c} C_{1}\frac{dv_{C_{1}}}{dt}=i_{L_{1}},\\ L_{1}\frac{di_{L_{1}}}{dt}=\left(k-1\right)\cdot R_{1}\cdot i_{L_{1}}-v_{C_{1}}-v_{C_{2}},\\ C_{2}\frac{dv_{C_{2}}}{dt}=i_{0}+i_{L_{1}}-i_{D}, \end{array}\right.$$

where $i_{D}$ is the current of the diode $D_{1}$; $i_{0}$ is the current of the resistor $R_{4}$; and *k* is the gain of the operational amplifier circuit. The system’s nonlinearity is introduced with the diode’s current:

(2) $$i_{D}=i_{S}\cdot \left[\exp\left(\frac{e\cdot v_{C_{2}}}{k_{B}\cdot T}\right)-1\right],$$

where *e* is the electron charge; *T* is the temperature; $i_{S}$ is the saturation current; and $k_{B}$ is the Boltzmann constant.

In our previous work [38], we obtained the discrete solution to the system (1) using Euler–Cromer numerical integration. The discrete system is expressed by the difference equations in (4).

(3) $$\begin{array}{ccc} x=\frac{v_{C_{1}}}{V_{T}}, & y=\frac{\rho \cdot i_{L_{1}}}{V_{T}}, & z=\frac{v_{C_{2}}}{V_{T}},\\ V_{T}=\frac{k_{B}T}{e}, & \rho =\sqrt{\frac{L_{1}}{C_{1}}}, & \varepsilon =\frac{C_{2}}{C_{1}},\\ a=\left(k-1\right)\frac{R_{1}}{\rho }, & b=\frac{\rho \cdot i_{0}}{V_{T}}, & c=\frac{\rho \cdot i_{S}}{V_{T}}. \end{array}$$

(4) $$\left\{\begin{array}{c} x_{n+1}=y_{n}\cdot \Delta \theta +x_{n},\\ y_{n+1}=\left(a\cdot y_{n}-x_{n}-z_{n}\right)\cdot \Delta \theta +y_{n},\\ z_{n+1}=\frac{\Delta \theta }{\varepsilon }\cdot \left(b+y_{n}-c\cdot \left(\exp\left(z_{n}\right)-1\right)\right)+z_{n}, \end{array}\right.$$

where a=0.5; b=5.769; $c=9.155\cdot 10^{-5}$; ε=0.15; and $\Delta \theta =2^{-10}$. It is important to note that in (3), *c* depends on the current $i_{S}$, meaning that this constant is variable. However, this constant is very small, and the original study [7] explicitly states that the chaotic mode is insensitive to *c*; thus, taking *c* as a constant is valid.

### 2.2. Vilnius Chaos Oscillator FPGA Implementation

This subsection describes the implementation of the Vilnius chaos oscillator difference Equation (4) in a digital design that is intended for an FPGA. Our previous work [38] explored the possibility of analog–discrete and discrete–analog synchronization using (4) implemented in MATLAB. This work implements (4) on an FPGA.

The first step of FPGA implementation is to outline the mathematical operations of (4) from the perspective of the sequential digital design. This is highlighted in (5).

(5) $$\left\{\begin{array}{c} \underset{\mathrm{Next}\;\mathrm{value}}{\underset{︸}{x_{n+1}}}=\underset{\mathrm{Current}\;\mathrm{value}}{\underset{︸}{x_{n}}}+\underset{\mathrm{Derivative}}{\underset{︸}{y_{n}}}\cdot \underset{\mathrm{Time}\;\mathrm{step}}{\underset{︸}{\Delta \theta }},\\ \underset{\mathrm{Next}\;\mathrm{value}}{\underset{︸}{y_{n+1}}}=\underset{\mathrm{Current}\;\mathrm{value}}{\underset{︸}{y_{n}}}+\underset{\mathrm{Derivative}}{\underset{︸}{\left(a\cdot y_{n}-x_{n}-z_{n}\right)}}\cdot \underset{\mathrm{Time}\;\mathrm{step}}{\underset{︸}{\Delta \theta }},\\ \underset{\mathrm{Next}\;\mathrm{value}}{\underset{︸}{z_{n+1}}}=\underset{\mathrm{Current}\;\mathrm{value}}{\underset{︸}{z_{n}}}+\underset{\mathrm{Derivative}}{\underset{︸}{\left(\frac{b}{\varepsilon }+\frac{1}{\varepsilon }\cdot y_{n}-\frac{c}{\varepsilon }\cdot \left(\exp\left(z_{n}\right)-1\right)\right)}}\cdot \underset{\mathrm{Time}\;\mathrm{step}}{\underset{︸}{\Delta \theta }}. \end{array}\right.$$

**Figure 2:**
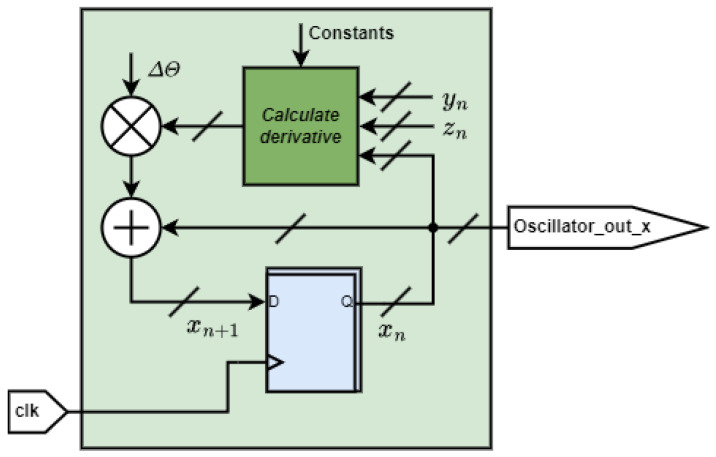
Digital design implementation of chaos oscillator system equations.

The equation clearly shows that each new value of the oscillator’s state variable is obtained by taking the current value and adding the derivative, which is a combination of state variables’ current states multiplied by a time step. This can be easily transferred to a digital circuit. Figure 2 demonstrates the circuit for the state variable *x*.

The circuit in the figure contains a register that, on the rising edge of the clock signal clk, registers the next value of the state variable *x*, thus acquiring the current state $x_{n}$ each clock cycle. The increment for the next state $x_{n+1}$ is obtained by passing the $x_{n}$, $y_{n}$, and $z_{n}$ values to the block calculating the derivatives. It processes all three state variables simultaneously, taking the current state-variable values and storing the constants from (5) required for the derivative calculations. For the case of *x*, this block will output $y_{n}$ as described in (5). Next, the derivative is multiplied by the time step Δθ. The multiplication result is added to the current state-variable value, $x_{n}$, thus obtaining $x_{n+1}$. The design performs similar operations to calculate updates for state variables *y* and *z*. The derivative calculation block is common for *x*, *y*, and *z*, with separate outputs for each state variable.

After establishing the general design for the digital implementation of the oscillator, the next step is designing the derivative calculation block, which is the core of the oscillator. The initial design choice was to utilize a pipeline approach. This means that the derivative calculation is split into simple mathematical operations followed by registers. The process requires the same delay for all results obtained in the pipeline when used for the particular operation. The pipelining process for the derivative calculation (5) is demonstrated in (6).

(6) $$\left\{\begin{array}{c} dx=\underset{1}{\underset{︸}{y_{n}}},\\ dy=\underset{2}{\underset{︸}{{\underset{︸}{a\cdot y_{n}}}_{1}-{\underset{︸}{x_{n}-z_{n}}}_{1}}},\\ dz=\underset{4}{\underset{︸}{\underset{3}{\underset{︸}{\underset{2}{\underset{︸}{\underset{1}{\underset{︸}{\frac{1}{\varepsilon }\cdot y_{n}}}-\frac{b}{\varepsilon }}}}}+\underset{3}{\underset{︸}{\frac{c}{\varepsilon }\cdot \underset{2}{\underset{︸}{\left(\underset{1}{\underset{︸}{\exp(z_{n})}}-1\right)}}}}}}. \end{array}\right.$$

For example, calculating dy would need to perform an $a\cdot y_{n}$ in the first clock cycle, while $x_{n}-z_{n}$ is done in parallel at the same clock cycle. After both results are registered, their sum is performed in the second clock cycle. Notably, the increment calculations of the state variables differ by the number of clock cycles required. However, oscillators must update all state variables simultaneously, which means that extra registers are required to match the delay of the longest pipeline, dz in this case.

Another point to consider is calculating the nonlinear function $\exp(z_{n})$. To obtain the exponent value in one clock cycle, we decided to use read-only memory (ROM) with the pre-calculated $\exp(z_{n})$ values for the possible $z_{n}$ range. This idea was further expanded by implementing the $-\frac{b}{\varepsilon }+\frac{c}{\varepsilon }\cdot \left(\exp\left(z_{n}\right)-1\right)$ in ROM, as it contains the aforementioned exponents and constants. This simplifies the pipeline, as shown in (7).

(7) $$\left\{\begin{array}{c} dx=\underset{1}{\underset{︸}{y_{n}}},\\ dy=\underset{2}{\underset{︸}{{\underset{︸}{a\cdot y_{n}}}_{1}-{\underset{︸}{x_{n}-z_{n}}}_{1}}},\\ dz=\underset{2}{\underset{︸}{{\underset{︸}{\frac{1}{\varepsilon }\cdot y_{n}}}_{1}+{\underset{︸}{\mathrm{ROM}(z_{n})}}_{1}}}. \end{array}\right.$$

The system was initially implemented in MATLAB to speed up the debugging process. The environment allows for manageable adjustment of the bit widths of the integer and fractional parts of the signals, which are required for fixed-point implementation on an FPGA. The ROM implementation is demonstrated for the MATLAB model; it is identical to the digital design.

**Figure 3:**
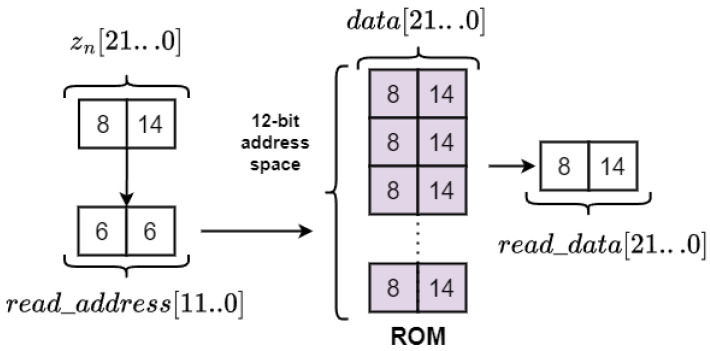
ROM design.

The design of the memory is shown in Figure 3. The bit widths of the integer and fractional parts are identical for the three state variables. The MATLAB model showed that $x_{n}$ requires at least 8 bits for the signed integer bit width. For this reason, the integer bit width was set to 8 bits for $x_{n}$, $y_{n}$, and $z_{n}$. According to MATLAB simulations, 14 bits are required for the fractional part, making the word length 22 bits. Figure 3 demonstrates that for the $z_{n}$, 6 integer part bits and 6 fractional part bits are used to form the ROM read address. Thus, the ROM is generated with a 12-bit address space, capable of storing 4096 data records, each consisting of 8 integer bits and 14 fractional bits.

**Figure 4:**
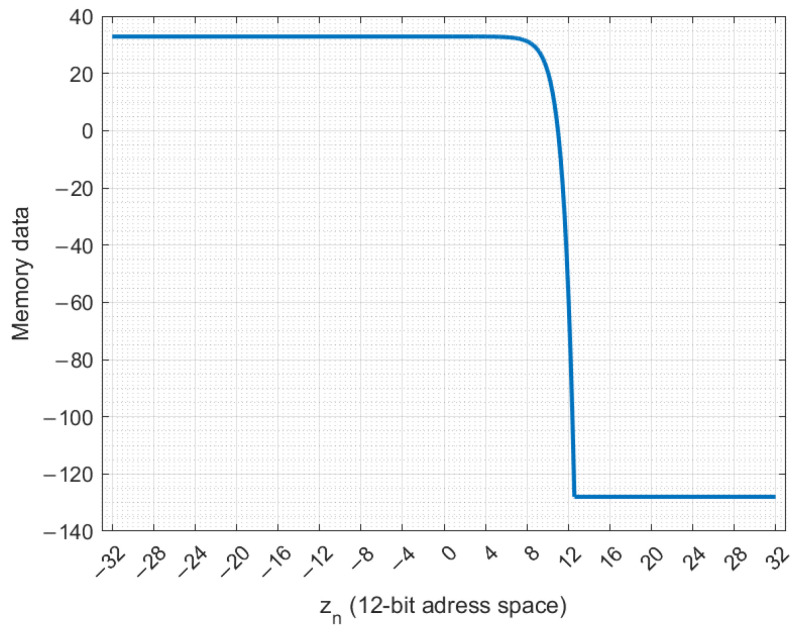
Memory data of $-\frac{b}{\varepsilon }+\frac{c}{\varepsilon }\cdot \left(\exp\left(z_{n}\right)-1\right)$ for the $z_{n}$ input range.

Figure 4 demonstrates the contents of the ROM in the case of the Vilnius chaos oscillator. The y-axis is used for the stored memory values of $-\frac{b}{\varepsilon }+\frac{c}{\varepsilon }\cdot \left(\exp\left(z_{n}\right)-1\right)$ for all possible read address ($z_{n}$ rounded to 12 bits) values on the x-axis. The input range is from −32 to 31.984375. For $z_{n}$ inputs greater than 12, the integer part of the in-memory expression exceeds 7 bits, so it is clipped to −128.

After establishing the complete digital design of the oscillator, including the block calculating the derivatives and the ROM storing the nonlinearity in MATLAB, the VHDL (Very High-Speed Integrated Circuit (VHSIC) Hardware Description Language (VHDL)) implementation was performed by translating the expressions.

### 2.3. RC Chaos Oscillator

**Figure 5:**
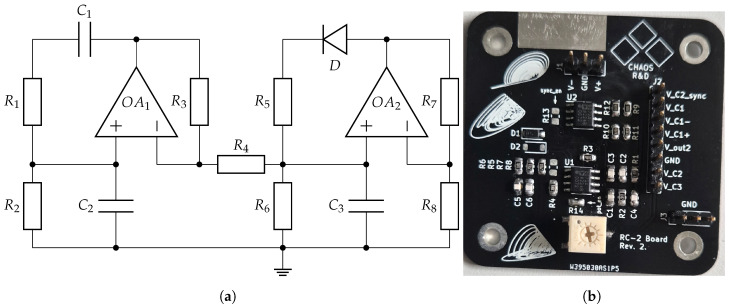
RC chaos oscillator [8] circuit (**a**) and PCB (**b**) with the following parameters: $C_{1}=C_{2}=C_{3}=1$ nF, $R_{1}=R_{2}=11$ kΩ, $R_{3}=9.1$ kΩ, $R_{4}=2$ kΩ, $R_{5}=R_{7}=2.7$ kΩ, $R_{6}=1.1$ kΩ, and $R_{8}=780$Ω. On the PCB $OA_{1}$ and $OA_{2}$ are an LT082 operational amplifier, and the diode is a 1N4148.

The second oscillator is the RC chaos oscillator [8]. The circuit of the oscillator is shown in Figure 5a, while the hardware implementation of the circuit using discrete components on an FR4 PCB is shown in Figure 5b.

System (8) describes the dynamics of this oscillator. The circuit’s state variables are the capacitor’s $C_{1},$$C_{2}$, and $C_{3}$ voltages. The nonlinearity in this case is modeled using the Heaviside step function *H*.

(8) $$\left\{\begin{array}{c} C_{1}\frac{dv_{C_{1}}}{dt}=-\frac{v_{C_{1}}}{R_{1}}-\frac{v_{C_{1}}-v_{C_{2}}}{R_{3}},\\ C_{2}\frac{dv_{C_{2}}}{dt}=\frac{v_{C_{1}}-v_{C_{2}}}{R_{3}}-\frac{v_{C_{2}}-v_{C_{3}}}{R_{5}},\\ C_{3}\frac{dv_{C_{3}}}{dt}=\frac{v_{C_{2}}-v_{C_{3}}}{R_{5}}-\frac{v_{C_{3}}}{R_{7}}+\frac{R_{1}}{R_{8}}\left(v_{C_{3}}-1\right)H\left(v_{C_{3}}-1\right). \end{array}\right.$$

The work in [8] presented Equation (9) by introducing the parameters shown in (10).

(9) $$\left\{\begin{array}{c} \frac{dx}{dt}=-x+\left(k_{1}-1\right)\cdot y-\left(k_{1}-1\right)\cdot z\\ \frac{dy}{dt}=-x+\left(k_{1}-2\right)\cdot y-\left(k_{1}-1\right)\cdot z\\ \frac{dz}{dt}=k_{1}\cdot y+\left(k_{1}+\alpha \right)\cdot z+\beta \cdot \left(z-1\right)\cdot H\left(z-1\right) \end{array}\right.$$

(10) $$\begin{array}{ccc} x=\frac{v_{C_{1}}}{v^{*}}, & y=\frac{v_{C_{2}}}{v^{*}}, & z=\frac{v_{C_{3}}}{v^{*}},\\ v^{*}=\frac{v_{0}}{k_{2}-1}, & \alpha =\frac{R_{1}}{R_{6}}, & \beta =\frac{R_{1}}{R_{8}},\\ k_{1}=\frac{R_{3}}{R_{4}}+1, & k_{2}=\frac{R_{7}}{R_{8}}+1, & \theta =\frac{1}{R_{1}\cdot C_{1}}. \end{array}$$

Similarly, [38] derived the discrete Equation (11) by applying Euler–Cromer numerical integration to (9).

(11) $$\left\{\begin{array}{c} x_{n+1}=\left(\left(k_{1}-1\right)\cdot y_{n}-\left(k_{1}-1\right)\cdot z_{n}-x_{n}\right)\cdot \Delta \theta +x_{n}\\ y_{n+1}=\left(\left(k_{1}-2\right)\cdot y_{n}-\left(k_{1}-1\right)\cdot z_{n}-x_{n}\right)\cdot \Delta \theta +y_{n}\\ z_{n+1}=\left(k_{1}\cdot y_{n}+\left(k_{1}+\alpha \right)\cdot z_{n}+\beta \cdot \left(z_{n}-1\right)\cdot H\left(z_{n}-1\right)\right)\cdot \Delta \theta +z_{n} \end{array}\right.$$

The final equation has the following parameters: $k_{1}=5.5$; α=10; β=14.1026; ε=0.15; and $\Delta \theta =2^{-10}$.

### 2.4. RC Chaos Oscillator FPGA Implementation

Similar to the Vilnius chaos oscillator, the first step in adapting the RC chaos oscillator’s state-variable differential Equation (11) to a digital design is to outline the mathematical operations from the perspective of a sequential digital design.

(12) $$\left\{\begin{array}{c} \underset{\mathrm{Next}\;\mathrm{value}}{\underset{︸}{x_{n+1}}}=\underset{\mathrm{Current}\;\mathrm{value}}{\underset{︸}{x_{n}}}+\underset{\mathrm{Derivative}}{\underset{︸}{\left(\left(k_{1}-1\right)\cdot y_{n}-\left(k_{1}-1\right)\cdot z_{n}-x_{n}\right)}}\cdot \underset{\mathrm{Time}\;\mathrm{step}}{\underset{︸}{\Delta \theta }}\\ \underset{\mathrm{Next}\;\mathrm{value}}{\underset{︸}{y_{n+1}}}=\underset{\mathrm{Current}\;\mathrm{value}}{\underset{︸}{y_{n}}}+\underset{\mathrm{Derivative}}{\underset{︸}{\left(\left(k_{1}-2\right)\cdot y_{n}-\left(k_{1}-1\right)\cdot z_{n}-x_{n}\right))}}\cdot \underset{\mathrm{Time}\;\mathrm{step}}{\underset{︸}{\Delta \theta }}\\ \underset{\mathrm{Next}\;\mathrm{value}}{\underset{︸}{z_{n+1}}}=\underset{\mathrm{Current}\;\mathrm{value}}{\underset{︸}{z_{n}}}+\underset{\mathrm{Derivative}}{\underset{︸}{\left(k_{1}\cdot y_{n}+\left(k_{1}+\alpha \right)\cdot z_{n}+\beta \cdot \left(z_{n}-1\right)\cdot H\left(z_{n}-1\right)\right)}}\cdot \underset{\mathrm{Time}\;\mathrm{step}}{\underset{︸}{\Delta \theta }} \end{array}\right.$$

The equation demonstrates that the system in (12) is very similar to (5) in the case of the Vilnius chaos oscillator. The only difference is the operations performed in the “derivative” part of the system. For this reason, it is possible to use the digital design presented in Figure 2, only adjusting the operations performed in the derivative calculation block. The next step is the pipeline implementation of the derivative calculation block. From (12), it can be seen that the nonlinearity (Heaviside function) has $z_{n}$ as an argument. Following the Vilnius oscillator implementation procedure described above, $\left(k_{1}+\alpha \right)\cdot z_{n}+\beta \cdot \left(z_{n}-1\right)\cdot H\left(z_{n}-1\right)$ was replaced with a ROM. The simplified pipeline is shown in (13).

(13) $$\left\{\begin{array}{c} dx=\underset{3}{\underset{︸}{{\underset{︸}{{\underset{︸}{\left(k_{1}-1\right)\cdot y_{n}}}_{1}-{\underset{︸}{\left(k_{1}-1\right)\cdot z_{n}}}_{1}}}_{2}-{\underset{︸}{{\underset{︸}{x_{n}}}_{1}}}_{2}}}\\ dy=\underset{3}{\underset{︸}{{\underset{︸}{{\underset{︸}{\left(k_{1}-1\right)\cdot y_{n}}}_{1}-{\underset{︸}{\left(k_{1}-2\right)\cdot z_{n}}}_{1}}}_{2}-{\underset{︸}{{\underset{︸}{x_{n}}}_{1}}}_{2}}}\\ dz=\underset{2}{\underset{︸}{{\underset{︸}{k_{1}\cdot y_{n}}}_{1}+{\underset{︸}{\mathrm{ROM}(z_{n})}}_{1}}} \end{array}\right.$$

.

**Figure 6:**
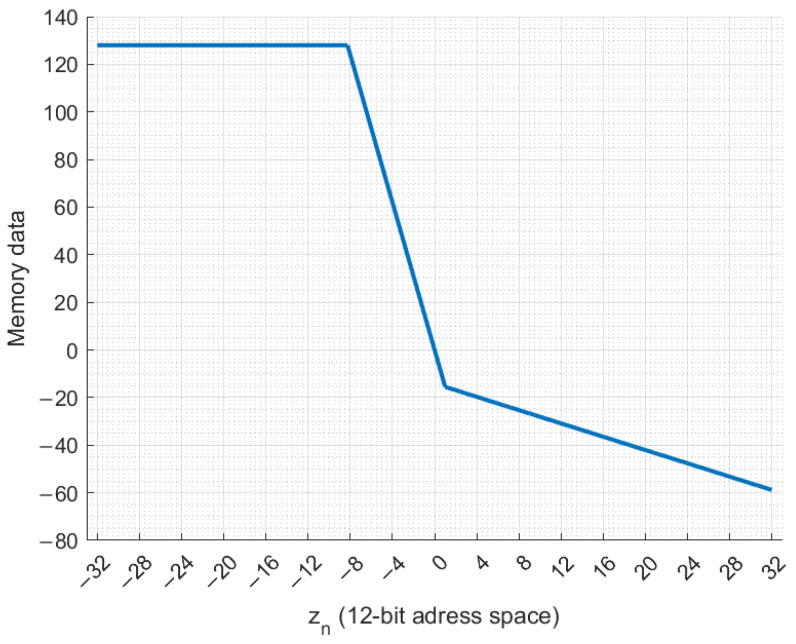
Memory data of $\left(k_{1}+\alpha \right)\cdot z_{n}+\beta \cdot \left(z_{n}-1\right)\cdot H\left(z_{n}-1\right)$ for the $z_{n}$ input range.

The ROM design is identical to that in Figure 3. The ROM contents for the RC chaos oscillator are shown in Figure 6. In this case, for $z_{n}<-8$, the in-memory expression values exceed the 8-bit signed integer value range, so the stored values are truncated to $128-2^{-14}$ (maximum signed 8-bit integer and 14-bit fractional parts fixed-point number).

Similarly, after establishing the complete digital design in MATLAB, the expressions were translated to VHDL for FPGA implementation.

### 2.5. MATLAB Model and VHDL Design Comparison

This subsection discusses the FPGA resource utilization and verifies the correspondence of the VHDL and MATLAB designs. A comparison was made between the two chaos oscillators.

The FPGA implementation was targeted at the Altera (Intel) Cyclone 5CSXFC6D6F31C6 chip on a DE10-Standard board. The utilization of FPGA resources for the VHDL design of the chaos oscillators is presented in Table 1. The FPGA resource utilization results demonstrate that the designed oscillators use only a small fraction of FPGA resources in their current form, meaning that the oscillators can be used in chips with fewer resources or in resource-intensive designs that may additionally utilize chaos oscillators. By comparing the two oscillators, the RC oscillator requires more adaptive logic modules (ALMs) and digital signal-processing (DSP) blocks, although the difference is minimal.

**Figure 7:**
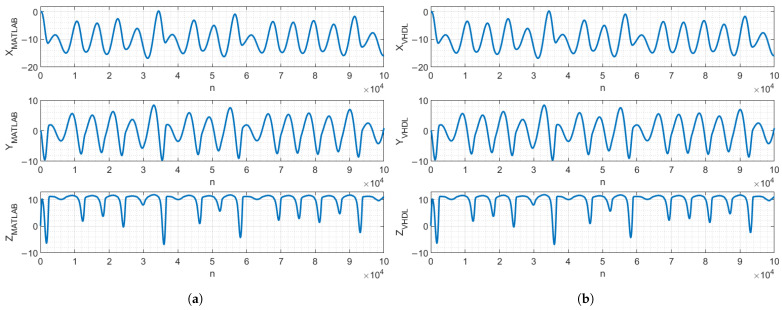
MATLAB (**a**) and VHDL-based digital design (**b**) of Vilnius chaos oscillator state-variable signals.

**Figure 8:**
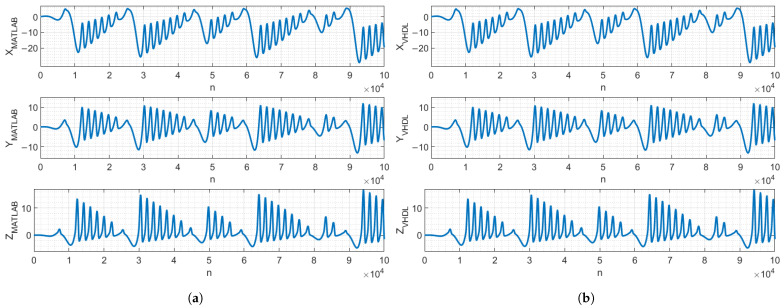
MATLAB (**a**) and VHDL-based digital design (**b**) of RC chaos oscillator state-variable signals.

To verify that the VHDL-based design performs identically to the MATLAB (2023) model, a functional simulation was performed using the Questa/Modelsim (2023.3) Intel FPGA simulation tool. The simulation results were exported to MATLAB for verification. Figure 7 and Figure 8 demonstrate that, with identical parameters and initial conditions, the chaos oscillators’ signals of the MATLAB model and VHDL-based design were identical and did not diverge in the $10^{5}$ clock cycles, *n*, meaning that the MATLAB model precisely described the behavior of the VHDL-based digital design.

## 3. Experimental Verification of FPGA-Based Analog–Discrete and Discrete–Analog Synchronization

**Figure 9:**
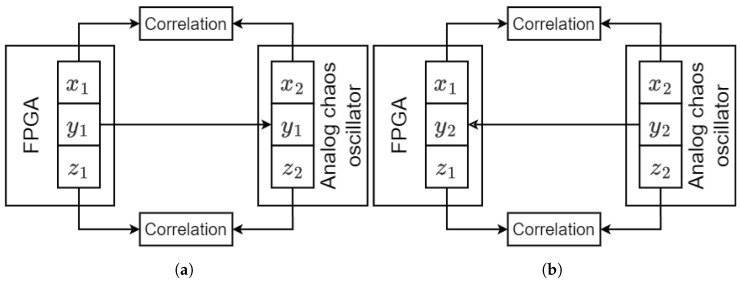
Discrete–analog (**a**) and analog–discrete (**b**) Pecora–Carroll synchronization.

This section is devoted to studying analog–discrete and discrete–analog synchronization using the chaos oscillators implemented on the PCB and FPGA-based chaos oscillators. The two chaos oscillators are asynchronous by default due to differences in initial conditions and mismatches in the parameters. The synchronization technique utilized to achieve analog–discrete and discrete–analog synchronization is known as Pecora–Carroll synchronization. This technique is depicted in Figure 9 for the two cases of chaotic synchronization. The state variables of the FPGA-based chaos oscillator are $x_{1}$, $y_{1}$, and $z_{1}$, while the state variables of the analog oscillator are $x_{2}$, $y_{2}$, and $z_{2}$.

In Figure 9a, the FPGA-based oscillator is a drive system, while the analog oscillator is a response system. Synchronization is achieved by passing the $y_{1}$ state variable to the response system and replacing $y_{2}$. By doing this, $x_{2}$ starts following the behavior of $x_{1}$, and $z_{2}$ starts following the behavior of $z_{1}$. Analog–discrete synchronization is shown in Figure 9b, where the analog oscillator is a drive system and the FPGA-based oscillator is a response system. By passing $y_{2}$ to the response system and replacing $y_{1}$, $x_{1}$ starts following the behavior of $x_{2}$, and $z_{1}$ starts following the behavior of $z_{2}$. The similarity of signals is evaluated by calculating the correlation of the corresponding signals. Our previous work [38] addressed analog–discrete and discrete–analog synchronization via modeling, whereas this work focuses on hardware.

**Figure 10:**
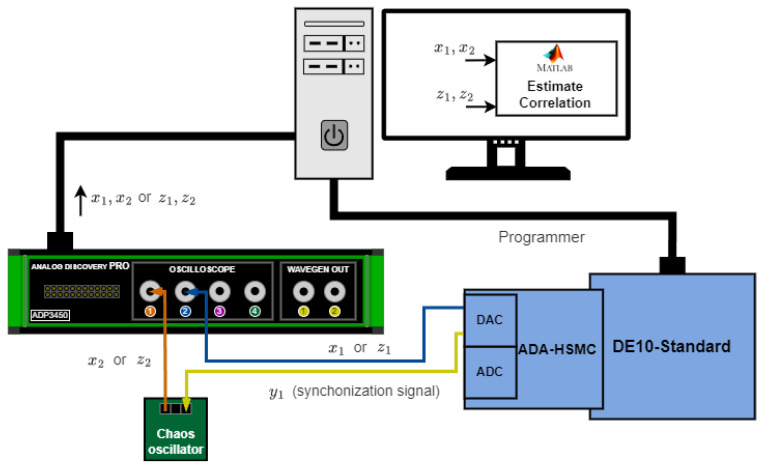
Experimental verification of discrete–analog chaotic synchronization.

Figure 10 demonstrates the experimental evaluation of discrete–analog synchronization. The FPGA-based chaos oscillator operates on a DE10-Standard board. An ADA-HSMC daughter card is connected via a High-Speed Mezzanine Card (HSMC) connector, providing a two-channel analog-to-digital converter (ADC) and a two-channel digital-to-analog converter (DAC). The first DAC channel outputs a $y_{1}$ synchronization signal applied to the analog chaos oscillator. The second DAC channel outputs $x_{1}$ in the first set of measurements and $z_{1}$ in the second set of measurements. ADP3450 oscilloscope channels are used to record and save $x_{1}$, $x_{2}$ and $z_{1}$, $z_{2}$. Next, a correlation between the corresponding signals is estimated in MATLAB.

**Figure 11:**
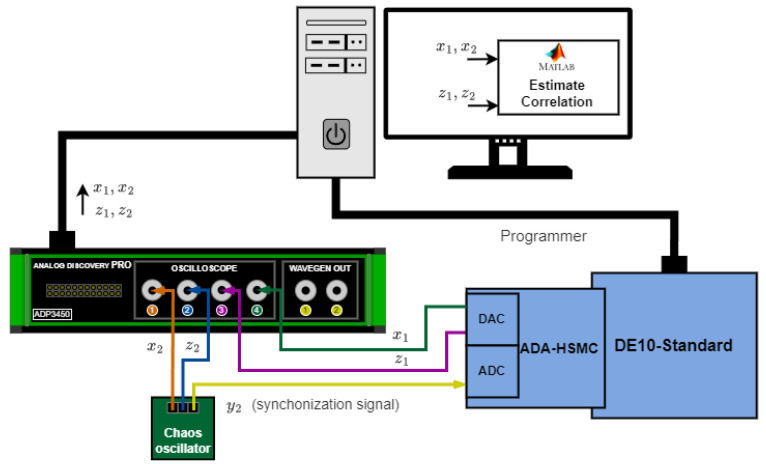
Experimental verification of analog–discrete chaotic synchronization.

Figure 11 demonstrates the experimental evaluation of analog–discrete synchronization. In this case, the $y_{2}$ signal is passed to the ADC input and forwarded to the FPGA-based oscillator. FPGA signals $x_{1}$ and $z_{1}$ are passed to the DAC. In this case, the ADP3450 oscilloscope channels record $x_{1}$, $x_{2}$ and $z_{1}$, $z_{2}$ simultaneously. The correlation is also estimated in MATLAB.

**Figure 12:**
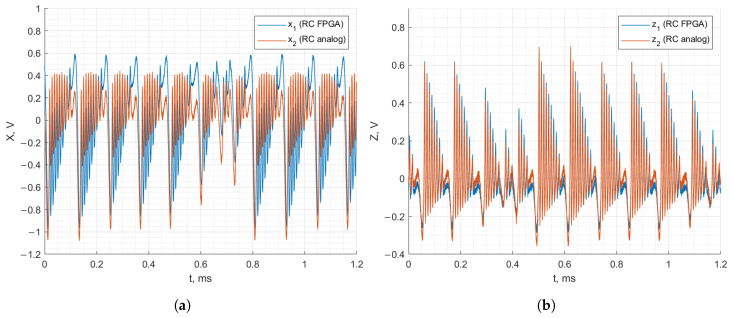
Discrete–analog synchronization of the RC chaos oscillator: *x* (**a**) and *z* (**b**) state variables.

**Figure 13:**
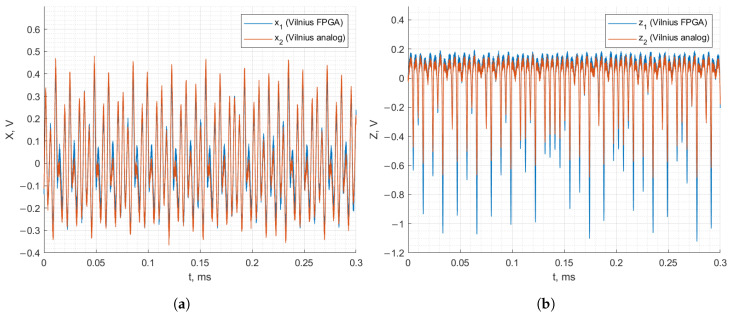
Discrete–analog synchronization of the Vilnius chaos oscillator: *x* (**a**) and *z* (**b**) state variables.

Figure 12 and Figure 13 demonstrate the recorded waveforms of the Vilnius and RC chaos oscillators with applied synchronization in the case of discrete–analog synchronization. The figures demonstrate that with the synchronization signal applied to the analog oscillator from the FPGA board, the behavior of the analog oscillator matches that of the FPGA-based oscillator. In the case of the Vilnius chaos oscillator, the $x_{1}$, $x_{2}$ and $z_{1}$, $z_{2}$ signals match almost perfectly. In the case of the RC chaos oscillator, it is important to note that the analog and FPGA-based oscillators are synchronized since $z_{1}$ and $z_{2}$ match, but the $x_{1}$ and $x_{2}$ signals do not coincide.

**Figure 14:**
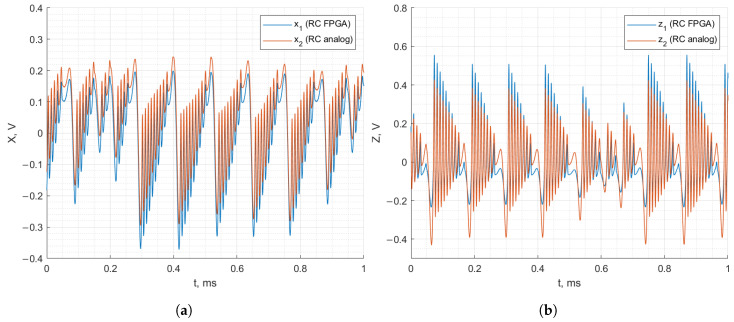
Analog–discrete synchronization of the RC chaos oscillator: *x* (**a**) and *z* (**b**) state variables.

**Figure 15:**
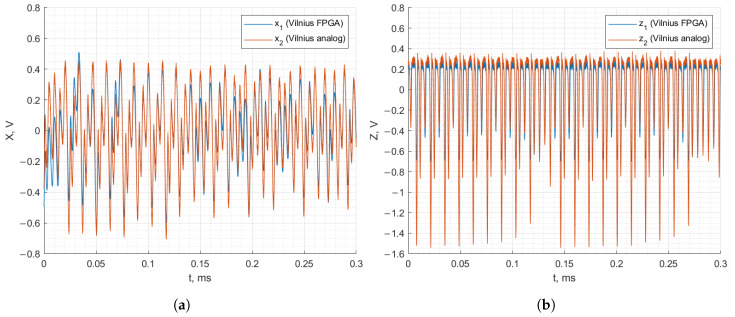
Analog–discrete synchronization of the Vilnius chaos oscillator: *x* (**a**) and *z* (**b**) state variables.

Figure 14 and Figure 15 demonstrate the recorded waveforms of the Vilnius and RC chaos oscillators with applied synchronization for analog–discrete synchronization. The figures demonstrate that, when the synchronization signal is applied to the FPGA-based oscillator from the analog oscillator, the FPGA-based oscillator’s behavior matches that of the analog oscillator. The $x_{1}$, $x_{2}$ and $z_{1}$, $z_{2}$ signals match between the two oscillators, demonstrating successful synchronization.

Table 2 presents the correlation coefficients calculated for every Vilnius and RC chaos oscillator’s state variable in the discrete–analog and analog–discrete synchronization cases. The correlation coefficient equaled 1 for identical signals, while 0 indicates that the signals were completely uncorrelated. The table shows that, in most cases, the correlation coefficient was over 0.9 and very close to 1, thus confirming that analog–discrete and discrete–analog synchronization were achieved between the analog and FPGA-based chaos oscillators. The fact that the signals did not coincide perfectly can be attributed to mismatched parameters in the analog and FPGA-based oscillators. Another contributing factor was noise.

## 4. Conclusions

This work focused on the FPGA implementation of analog chaos oscillators for analog–discrete and discrete–analog chaotic synchronization applications. The key achievement of this work is the experimentally verified analog–discrete and discrete–analog synchronization between the FPGA-based and analog chaos oscillators. This work developed an approach for the parallel implementation of such chaos oscillators on an FPGA, allowing the application of the technique to other chaos oscillators and integrating the oscillators into FPGA-based designs. Additionally, the developed FPGA implementation approach demonstrated acceleration by using ROM for nonlinearity approximation. This achievement also enables the use of the FPGA to modify the parameters of the chaos oscillators, increasing the oscillation frequency and changing the signal bandwidths. The developed precise MATLAB model of the digital design allows for studying the performance of the FPGA-based oscillators without the need for hardware, as well as testing and debugging design ideas for the FPGA-based oscillators.

Regarding analog–discrete and discrete–analog synchronization, the synchronization pattern for the RC chaos oscillator was observed previously, where the shape of the *x* state variables did not perfectly match in the case of discrete–analog synchronization; however, the correlation in this case was sufficiently high. The achieved analog–discrete and discrete–analog synchronization of the analog and FPGA-based oscillators introduces new possibilities for chaos-based data transmission, as the systems can now utilize both types of oscillators, creating a hybrid analog–discrete chaos-based transmission system.

## Figures and Tables

**Table 1:** FPGA resource utilization of chaos oscillators.

Chaos Oscillator	Adaptive Logic Modules (ALMs)	Digital Signal-Processing (DSP) Blocks	Block Memory Bits
Vilnius	67 (<1%)	1 (1%)	90,122 (2%)
RC	115 (<1%)	5 (6%)	90,122 (2%)

**Table 2:** Synchronization evaluation using correlation coefficient.

	Discrete–Analog		Analog–Discrete	
Chaos Oscillator	$x_{1}$ and $x_{2}$	$z_{1}$ and $z_{2}$	$x_{1}$ and $x_{2}$	$z_{1}$ and $z_{2}$
Vilnius	0.97	0.98	0.93	0.92
RC	0.72	0.97	0.89	0.90

## Data Availability

The original contributions presented in this study are included in the article. Further inquiries can be directed to the corresponding author(s). The MATLAB models and VHDL implementation of the oscillators are available in the following repository: https://github.com/RBabajans/MATLAB_model_and_VHDL_implementation_of_chaos_oscillators (accessed on 20 March 2025).

## Abbreviations

The following abbreviations are used in this manuscript:

ALM	Adaptive logic module
ADC	Analog-to-digital converter
DAC	Digital-to-analog converter
DSP	Digital signal processing
FPGA	Field-programmable gate array
HSMC	High-Speed Mezzanine Card
IoT	Internet of Things
PCB	Printed circuit board
ROM	Read-only memory
VHDL	VHSIC Hardware Description Language
VHSIC	Very High-Speed Integrated Circuit
WSN	Wireless Sensor Network

## References

[1] (2024). Ericsson Mobility Report November 2024.

[2] El Hanine M., El-Yahyaoui A., Es-Sadaoui R. Three Layer IoT Architecture: Attacks and Security Mechanisms. Proceedings of the 2024 11th International Conference on Future Internet of Things and Cloud (FiCloud).

[3] Frustaci M., Pace P., Aloi G., Fortino G. (2018). Evaluating Critical Security Issues of the IoT World: Present and Future Challenges. IEEE Internet Things J..

[4] Neshenko N., Bou-Harb E., Crichigno J., Kaddoum G., Ghani N. (2019). Demystifying IoT Security: An Exhaustive Survey on IoT Vulnerabilities and a First Empirical Look on Internet-Scale IoT Exploitations. IEEE Commun. Surv. Tutor..

[5] Chua L., Wu C., Huang A., Zhong G.-Q. (1993). A universal circuit for studying and generating chaos. I. Routes to chaos. IEEE Trans. Circuits Syst. I Fundam. Theory Appl..

[6] Kennedy M. (1994). Chaos in the Colpitts oscillator. IEEE Trans. Circuits Syst. I Fundam. Theory Appl..

[7] Tamaševičius A., Mykolaitis G., Pyragas V., Pyragas K. (2004). A simple chaotic oscillator for educational purposes. Eur. J. Phys..

[8] Namajunas A., Tamasevicius A. (1996). Simple RC chaotic oscillator. Electron. Lett..

[9] Chen X., Du H., Luo J., Chen Y. Memristor-based chaotic synchronization control and circuit experiments via single-input controller. Proceedings of the 2024 36th Chinese Control and Decision Conference (CCDC).

[10] Escudero M., Spiga S., Marco M.D., Forti M., Innocenti G., Tesi A., Corinto F., Brivio S. (2024). Chua’s Circuit with Tunable Nonlinearity Based on a Nonvolatile Memristor: Design and Realization. IEEE Trans. Circuits Syst. I Regul. Pap..

[11] Chen W.L., Zheng L.H., Song X.X. Design of two-stage chaotic Colpitts oscillator. Proceedings of the 2016 IEEE International Conference on Microwave and Millimeter Wave Technology (ICMMT).

[12] Semenov A.O., Savytskyi A.Y., Bisikalo O.V., Kulakov P.I. Mathematical modeling of the two-stage chaotic colpitis oscillator. Proceedings of the 2018 14th International Conference on Advanced Trends in Radioelecrtronics, Telecommunications and Computer Engineering (TCSET).

[13] Petrzela J., Kaller O., Vavra J. (2024). The Reinartz Oscillator: Analysis Beyond Regular Behavior of the Circuit. IEEE Access.

[14] Sadia M., Paul P.S., Hossain M.R., Muldrey B., Hasan M.S. (2023). Robust Chaos with Novel 4-Transistor Maps. IEEE Trans. Circuits Syst. II Express Briefs.

[15] Jiang Y., Li C., Zhang C., Lei T., Jafari S. (2023). Constructing Meminductive Chaotic Oscillator. IEEE Trans. Circuits Syst. II Express Briefs.

[16] Banerjee T., Karmakar B., Sarkar B.C. (2012). Chaotic electronic oscillator from single amplifier biquad. AEU Int. J. Electron. Commun..

[17] Wang L., Wang C., Zhang H., Ma P., Zhang S. (2024). Estimation-Correction Modeling and Chaos Control of Fractional-Order Memristor Load Buck-Boost Converter. Complex Syst. Model. Simul..

[18] Benadero L., Aroudi A.E., Martínez-Salamero L., Tse C.K. Period Doubling Route to Chaos in Open Loop Boost Converters under Constant Power Loading and Discontinuous Conduction Mode Conditions. Proceedings of the 2020 IEEE International Symposium on Circuits and Systems (ISCAS).

[19] Zhang C., Zhang S., Liang K., Chen Z. (2024). Double Image Encryption Algorithm Based on Parallel Compressed Sensing and Chaotic System. IEEE Access.

[20] Kumar Thukral M. SCLLCM: A Robust One Dimesional Chaotic Map for Image Encryption Application. Proceedings of the 2024 Asia Pacific Conference on Innovation in Technology (APCIT).

[21] Atteya A.M., Madian A.H. A hybrid Chaos-AES encryption algorithm and its impelmention based on FPGA. Proceedings of the 2014 IEEE 12th International New Circuits and Systems Conference (NEWCAS).

[22] Moysis L., Kafetzis I., Volos C., Tutueva A.V., Butusov D. Application of a Hyperbolic Tangent Chaotic Map to Random Bit Generation and Image Encryption. Proceedings of the 2021 IEEE Conference of Russian Young Researchers in Electrical and Electronic Engineering (ElConRus).

[23] Butusov D.N., Karimov T.I., Lizunova I.A., Soldatkina A.A., Popova E.N. Synchronization of analog and discrete Rössler chaotic systems. Proceedings of the 2017 IEEE Conference of Russian Young Researchers in Electrical and Electronic Engineering (EIConRus).

[24] Zhang J., Qi L. Detection of Weak Signals Based on the Duffing Chaotic System and FPGA Implementation. Proceedings of the 2024 5th International Seminar on Artificial Intelligence, Networking and Information Technology (AINIT).

[25] Bonny T., Al Nassan W. (2024). NeuroChaosCrypt: Revolutionizing Chaotic-Based Cryptosystem with Artificial Neural Networks—A Comparison with Traditional Cryptosystems. IEEE Access.

[26] Addabbo T., Fort A., Moretti R., Mugnaini M., Takaloo H., Vignoli V. A New Class of Chaotic Sources in Programmable Logic Devices. Proceedings of the 2020 IEEE International Workshop on Metrology for Industry 4.0 & IoT.

[27] Karataş O., Demir K., Ergün S. Chaotic Sampling of Double Scroll Chaos for Digital Random Number Generation. Proceedings of the 2022 IEEE Asia Pacific Conference on Circuits and Systems (APCCAS).

[28] Azzaz M.S., Kaibou R., Smahi A. FPGA Implementation using Novel Co-Design Approach of Real-Time Speech Chaos based Crypto-Watermarking Prototype. Proceedings of the 2023 International Conference on Advances in Electronics, Control and Communication Systems (ICAECCS).

[29] Hobincu R., Datcu O. FPGA Implementation of a Chaos Based PRNG Targetting Secret Communication. Proceedings of the 2018 International Symposium on Electronics and Telecommunications (ISETC).

[30] Azzaz M.S., Tanougast C., Adoudi S., Bouridane A., Dandache A. An FPGA implementation of a Feed-Back Chaotic Synchronization for secure communications. Proceedings of the 2010 7th International Symposium on Communication Systems, Networks and Digital Signal Processing, CSNDSP 2010.

[31] Babu R.R., Karthikeyan R. Adaptive synchronization of novel chaotic system and its FPGA implementation. Proceedings of the 2015 International Conference on Smart Technologies and Management for Computing, Communication, Controls, Energy and Materials, ICSTM 2015.

[32] Guillén-Fernández O., Meléndez-Cano A., Tlelo-Cuautle E., Núñez-Pérez J.C., de Jesus Rangel-Magdaleno J. (2019). On the synchronization techniques of chaotic oscillators and their FPGA-based implementation for secure image transmission. PLoS ONE.

[33] Kaddoum G. (2016). Wireless Chaos-Based Communication Systems: A Comprehensive Survey. IEEE Access.

[34] Kvitko D., Babkin I., Shirnin K., Karimov T., Kolev G., Rybin V. Chaos Shift Keying Coherent Communication Based on Different Types of Operational Amplifiers. Proceedings of the 2023 12th Mediterranean Conference on Embedded Computing (MECO).

[35] George A.E., Gelóczi E., Mexis N., Arul T., Katzenbeisser S., Stavrinides S.G., Picos R., Anagnostopoulos N.A. Real-World Secure Communication based on Synchronised Lorenz Chaotic Circuits. Proceedings of the 2024 13th International Conference on Modern Circuits and Systems Technologies (MOCAST).

[36] Han T., Dou X., Lin B. A Non-orthogonal Multi-carrier Frequency Hopping Based Secure Differential Chaos Shift Keying for Autonomous Ummanned Systems. Proceedings of the 2024 IEEE/CIC International Conference on Communications in China (ICCC Workshops).

[37] Kennedy M., Kolumban G., Kis G., Jako Z. (2000). Performance evaluation of FM-DCSK modulation in multipath environments. IEEE Trans. Circuits Syst. I Fundam. Theory Appl..

[38] Babajans R., Cirjulina D., Capligins F., Kolosovs D., Litvinenko A. (2024). Synchronization of Analog-Discrete Chaotic Systems for Wireless Sensor Network Design. Appl. Sci..

[39] An Y.H., Xu H., Zhang Y.Y., Wang H.Z., Xie K.Y., Zhang C.K. Design and implementation of master-slave synchronization for chaotic Lur’e systems using Chua’s circuit. Proceedings of the 2024 43rd Chinese Control Conference (CCC).

[40] Cheng H., Li H., Dai Q., Yang J. (2023). A deep reinforcement learning method to control chaos synchronization between two identical chaotic systems. Chaos Solitons Fractals.

[41] Chou H.G., Chuang C.F., Wang W.J., Lin J.C. (2013). A fuzzy-model-based chaotic synchronization and its implementation on a secure communication system. IEEE Trans. Inf. Forensics Secur..

[42] Du L., Wang F., Han Z., Dong J. Chaos Synchronization of a Class of Chaotic Systems via Linear State Error Feedback Control. Proceedings of the 2nd International Conference on Advances in Mechanical Engineering and Industrial Informatics (AMEII 2016).

[43] Bendoukha S., Abdelmalek S., Ouannas A. (2019). Secure communication systems based on the synchronization of chaotic systems. Stud. Syst. Decis. Control.

[44] Bilgehan B., Sabuncu O. Synchronization and Analysis of Chaotic Circuit with Application to Communication in the internet of things (IoT) Services. Proceedings of the 2022 International Conference on Artificial Intelligence in Everything (AIE).

[45] Çiçek S., Kocamaz U.E., Uyaroğlu Y. (2019). Secure Chaotic Communication with Jerk Chaotic System Using Sliding Mode Control Method and Its Real Circuit Implementation. Iran. J. Sci. Technol. Trans. Electr. Eng..

[46] Deniz H.I., Gulru Cam Taskiran Z., Sedef H. Chaotic Lorenz Synchronization Circuit Design for Secure Communication. Proceedings of the 2018 6th International Conference on Control Engineering & Information Technology (CEIT).

[47] Gularte K.H., Gomez J.C., Vizcarra Melgar M.E., Vargas J.A. Chaos Synchronization and its Application in Parallel Cryptography. Proceedings of the 2021 IEEE 5th Colombian Conference on Automatic Control (CCAC).

[48] Karimov T., Butusov D., Andreev V., Karimov A., Tutueva A. (2018). Accurate Synchronization of Digital and Analog Chaotic Systems by Parameters Re-Identification. Electronics.

